# Portal Vein Thrombosis, Management and Approaches in Liver Transplantation: A Narrative Review

**DOI:** 10.3390/jcm14176100

**Published:** 2025-08-28

**Authors:** João Bernardo Sancio, Raul Valério Ponte, Thiago Beduschi, Daiki Soma

**Affiliations:** 1 Department of Surgery, Faculty of Medicine, Federal University of Minas Gerais, Belo Horizonte 31270-901, MG, Brazil; joaosancio@ufmg.br; 2 Department of General Surgery, Dr. José Frota Institute, Fortaleza 60025-061, CE, Brazil; rppp@live.com; 3 Department of Surgery, Division of Transplantation & Hepatobiliary Surgery, University of Florida, Gainesville, FL 32610, USA

**Keywords:** portal vein thrombosis, thrombus, liver transplantation, anticoagulation, cirrhosis

## Abstract

This review delves into the complexities of managing portal vein thrombosis (PVT) in the context of liver transplantation (LT). PVT, which is a common finding in cirrhotic livers, can significantly jeopardize LT outcomes. Here, we explore the incidence, underlying mechanisms, and comprehensive management strategies for PVT throughout the pre-, intra-, and postoperative phases of LT in cirrhotic patients. Before transplantation, key interventions include anticoagulation therapies, transjugular intrahepatic portosystemic shunts (TIPS), and various endovascular techniques aimed at recanalizing the portal vein. During LT, surgical approaches range from straightforward eversion thrombectomy to more intricate procedures, such as jump grafts from the superior mesenteric vein (SMV), renoportal anastomosis (RPA), and portocaval hemitransposition, tailored to the extent of the thrombosis. In cases of extensive PVT, multivisceral transplantation (MVT) emerges as a viable option. Post-transplant management centers on thromboprophylaxis and anticoagulation, balancing the prevention of thrombotic events with the risk of bleeding complications. This review underscores the critical importance of early identification and proactive management of PVT to enhance outcomes for LT candidates.

## 1. Introduction

Liver transplantation (LT) is considered the treatment of choice for decompensated liver cirrhosis and offers the possibility of cure for select patients. LT can effectively reverse portal hypertension. Portal vein thrombosis (PVT) is commonly observed in patients with portal hypertension due to liver cirrhosis. PVT can significantly increase the risks of mortality and morbidity associated with LT, and in some cases, may preclude LT. Extensive PVT impairs portal vein inflow toward the liver graft and leads to worse graft survival [1]. Early diagnosis of PVT and appropriate management are key to achieving successful LT. This review addresses the mechanisms, incidence, diagnosis, and the management of pre-, intra-, and postoperative care of cirrhotic patients with PVT.

## 2. Incidence and Mechanisms

The prevalence of PVT in cirrhosis ranges from 5% to 26%, with most cases presenting as partial, non-obstructive thrombosis [2]. It is important to note that, despite pretransplant imaging studies, PVT is frequently identified intraoperatively. As emphasized by Ghabril et al. in a retrospective analysis of nearly fifty thousand transplant recipients, about 70% of PVT cases were only detected during transplantation [3]. Another study found a 15.7% prevalence of non-tumoral PVT in a retrospective analysis of approximately one thousand patients. A significantly higher prevalence of PVT was observed in patients with hepatitis B virus infection (*p* = 0.01), hepatocellular carcinoma (*p* < 0.01), ascites (*p* = 0.01), and spontaneous bacterial peritonitis (*p* = 0.02). Additionally, patients with thrombosis had higher international normalized ratio (INR) levels (*p* = 0.042), lower serum albumin values (*p* = 0.01), and higher MELD (*p* = 0.01) and Child-Pugh scores (*p* = 0.03) [4].

Chronic liver disease involves a series of coagulation abnormalities, creating a complex imbalance caused by reduced synthesis of coagulation factors, defective fibrinolytic activity, and platelet dysfunction. Although this hepatic dysfunction may appear to be “protective” against thrombotic events, patients experience a decrease in both procoagulant and anticoagulant factors, resulting in a delicate hemostatic balance [5]. A higher frequency of inherited thrombophilic disorders has been described in cirrhotic patients with PVT compared to those with no PVT, such as mutations in the prothrombin gene (G20210A) and factor V Leiden (FVL). Moreover, these patients also tend to show a progressive reduction in natural coagulation inhibitors over time, such as protein C, protein S, and antithrombin, leading to a hypercoagulable state [6,7,8].

Anatomical and hemodynamic factors are also described as predisposing conditions for PVT. Increased hepatic resistance secondary to cirrhosis leads to a reduction in portal venous flow, which has been considered one of the main factors associated with the development of PVT in these patients. A prospective study identified a portal flow velocity less than 15 cm/s on initial Doppler ultrasound as the strongest predictive factor for PVT development in cirrhotic patients (OR 44.9, 95% CI 5.3–382.7; *p* < 0.001) [8].

## 3. Portal Vein Thrombosis Classification

PVT can be subdivided according to their evolution into acute or chronic. Understanding the extent of portal thrombosis is crucial for the surgical planning of liver transplantation or even for evaluating its feasibility, since we can observe from small partial thrombi exclusive to the portal vein to diffuse thromboses extending throughout the splenic system [9].

**Figure 1:**
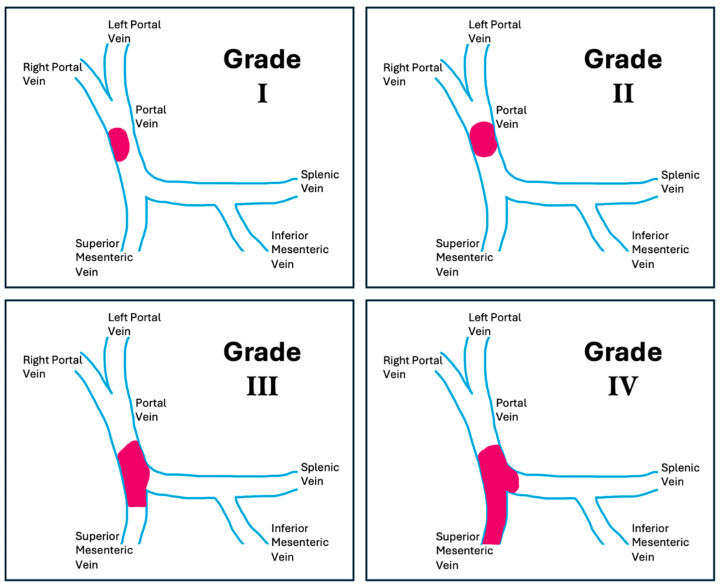
Yerdel Classification of PVT. The portal and mesenteric venous system is shown in blue, and the thrombus is highlighted in pink.

Specifically in the context of liver transplantation, the most widely used classification for surgical planning was proposed by Yerdel and colleagues (Table 1, Figure 1) [10].

## 4. Management of Portal Vein Thrombosis in Candidates for Transplantation

Since PVT can have a direct impact on morbidity and mortality in patients on the transplant waiting list, its early identification and management are crucial to reducing transplantation-related risks. The main goal of management is to achieve portal vein recanalization or, at a minimum, to prevent thrombus progression, as the advancement of thrombosis may contraindicate LT or require alternative portal reconstructions, which are associated with increased morbidity and mortality [11].

Physiological portal reconstruction—restoring normal portal venous flow to the graft—is strongly preferred whenever feasible, as it is associated with better graft function, lower incidence of complications, and improved patient survival compared to non-physiological alternatives [10,11,12]. However, this is not always possible in cases of extensive thrombosis.

The main therapeutic options for managing PVT in the pretransplant setting include the use of anticoagulants, transjugular intrahepatic portosystemic shunt (TIPS), and other endovascular interventions.

### 4.1. Anticoagulation in Pre-Transplant Candidates

The management of anticoagulation in patients with portal vein thrombosis requires careful attention, as it is necessary to balance the risk of bleeding with that of thrombus progression, especially in patients with a history of variceal bleeding. Importantly, evidence shows that successful anticoagulation is associated with lower rates of decompensation and improved survival in cirrhotic patients [12].

According to the Baveno VI consensus, anticoagulation is recommended for potential liver transplant candidates with main trunk portal vein thrombosis or progressive thrombosis, to facilitate transplantation and reduce post-transplant morbidity and mortality [13]. These recommendations are based on expert consensus and observational studies.

Low-molecular-weight heparin is generally preferred, as it appears to prevent both portal vein thrombosis and hepatic decompensation. Warfarin and direct oral anticoagulants (DOACs) may also be used [12,14]. Warfarin is the most used oral anticoagulant, but its interaction with food and the need for INR monitoring can complicate adherence. Additionally, cirrhotic patients often have elevated baseline INR, making dosing more challenging.

DOACs are considered promising alternatives, as they do not require laboratory dose adjustment and offer better adherence. According to some observational studies and systematic reviews, DOACs may provide higher rates of recanalization and lower rates of thrombosis extension compared to warfarin. They also appear to be non-inferior in terms of safety and efficacy [14]. However, current guidelines do not yet provide strong recommendations for the routine use of DOACs in this setting, and further high-quality studies are needed to confirm these findings.

### 4.2. Transjugular Intrahepatic Portosystemic Shunt (TIPS)

TIPS aims to restore low-resistance portal flow by creating an artificial shunt connecting the portal and hepatic veins. By restoring this flow and decompressing the portal venous system, the risk of complications secondary to portal hypertension, such as digestive hemorrhages and ascites, is significantly reduced. Moreover, studies have shown that TIPS can promote portal vein recanalization in cirrhotic patients with PVT [15,16].

A meta-analysis that evaluated the results of 18 studies, mostly retrospective and observational, showed a portal vein recanalization rate of 84.4% (95% CI = 78.4–89.0%), with complete recanalization in 73.7% of cases. The rate of associated encephalopathy was 25.3% [17].

Another study following 43 cirrhotic patients with PVT observed complete resolution of main portal vein thrombosis in 76% of cases without the use of associated anticoagulation, as seen in follow-up venography one month after TIPS placement [18].

This was also found by Wang and colleagues in a Chinese prospective randomized trial, which aimed to evaluate whether the addition of post-TIPS anticoagulation in this population would alter portal vein patency status and clinical outcomes during the first-year follow-up after TIPS. The study found no significant differences between the groups analyzed (TIPS + anticoagulation vs. TIPS without anticoagulation) regarding portal vein patency or clinical outcomes, suggesting that anticoagulant use after the installation of an artificial shunt may not be necessary [19].

It is important to underline that, especially from the perspective of liver transplantation, having an experienced interventional radiology team is crucial to avoid TIPS misplacement and surgical complications [20]. Advances in TIPS studies and results in cirrhotic patients with PVT have provided safety and effectiveness, allowing for physiological portal reconstruction with end-to-end anastomosis during liver transplantation [21]. The predominant mechanism by which TIPS promotes portal vein recanalization appears to be the restoration of portal venous flow. Increased flow through the shunt facilitates endogenous thrombolysis and remodeling of the native portal vein, leading to long-term patency and spontaneous resolution of thrombus. Thus, the process is primarily flow-mediated, as relieving stasis and restoring physiological hemodynamics are critical for thrombus resolution [21].

Although TIPS is effective for many patients, it is not suitable for all anatomical patterns of PVT or for patients with advanced liver failure, severe hepatic encephalopathy, or significant cardiac comorbidities. Careful patient selection remains essential.

## 5. Strategies During Liver Transplantation

Adequate identification and stratification of portal vein thrombosis (PVT) in the pre-transplant setting are essential for proper surgical planning. Doppler ultrasound of the liver is a non-invasive, widely available, and low-cost method. However, despite its ability to characterize portal anatomy and assess potential vascular shunts, it is an examiner-dependent method that may underestimate the diagnosis of thrombosis and is also challenging for thrombosis classification [22].

Multidetector computed tomography (MDCT) is the primary imaging modality used for assessing portal anatomy, classifying/staging PVT, and guiding surgical planning by defining potential alternatives for portal reconstruction. With sensitivity and specificity of 82% and 100%, respectively, MDCT also allows differentiation between tumor and non-tumor thrombi [1,23].

Portography is an invasive contrast-enhanced study that can be valuable both preoperatively and intraoperatively, aiding in PVT stratification as well as providing a dynamic evaluation of the portal system and its collaterals to identify potential portosystemic shunts. Intraoperatively, this method not only facilitates thrombosis identification but also guides interventions such as collateral ligation or endovascular treatments, including portal vein stent placement [24,25].

**Figure 2:**
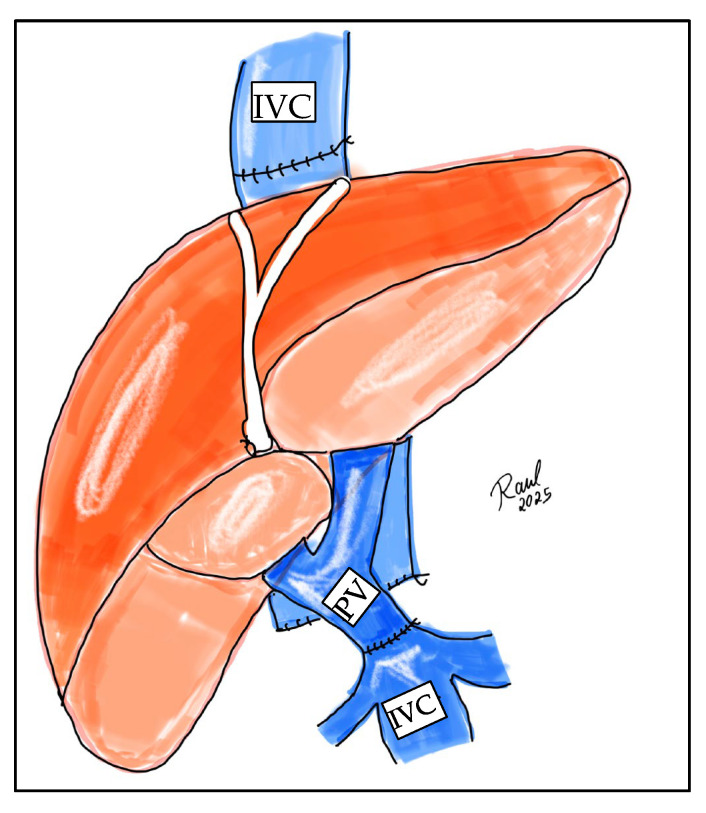
Illustration of portocaval hemitransposition. The liver parenchyma is shown in orange, the inferior vena cava (IVC) in blue, and the portal vein (PV) in blue. The recipient inferior vena cava is used for portal inflow to the allograft, performing a direct end-to-end side anastomosis with the donor portal vein (PV).

**Figure 3:**
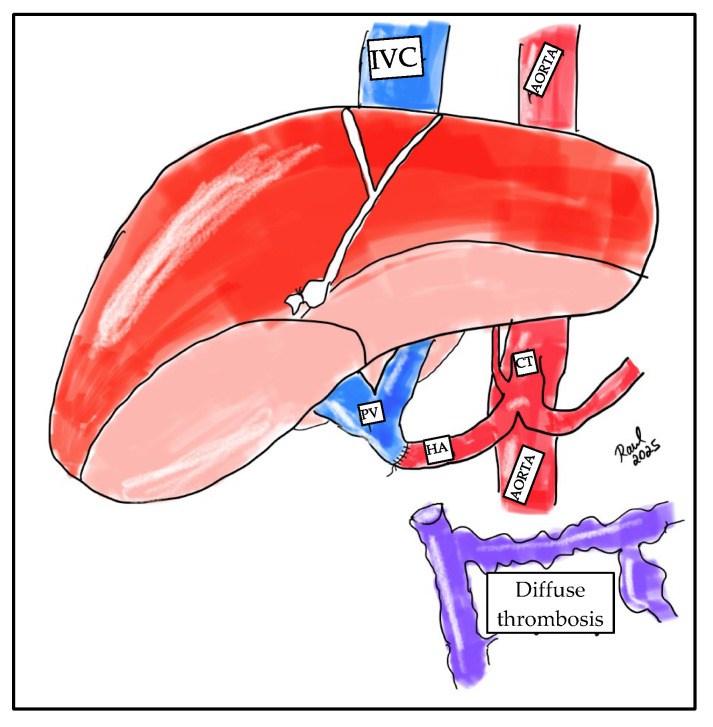
Illustration of portal vein arterialization. The liver parenchyma is shown in red, the inferior vena cava (IVC) in blue, the donor portal vein (PV) in blue, and the celiac trunk (CT) in red. Diffuse thrombosis of the portal mesenteric axis is represented in purple. HA: recipient hepatic artery. The schematic also shows a complete thrombosis of the portal mesenteric axis.

Ideally, the surgical strategy should aim for physiological portal reconstruction, in which portal circulation is drained to systemic circulation, such as in cases of thrombectomy with end-to-end portal anastomosis or the use of venous graft interpositions (mesoportal jump grafts). Conversely, non-physiological portal reconstructions are those in which normal portal flow through the liver cannot be reestablished, as seen in cavoportal hemitransposition (Figure 2), or portal vein arterialization (Figure 3).

### 5.1. Eversion Thrombectomy

**Figure 4:**
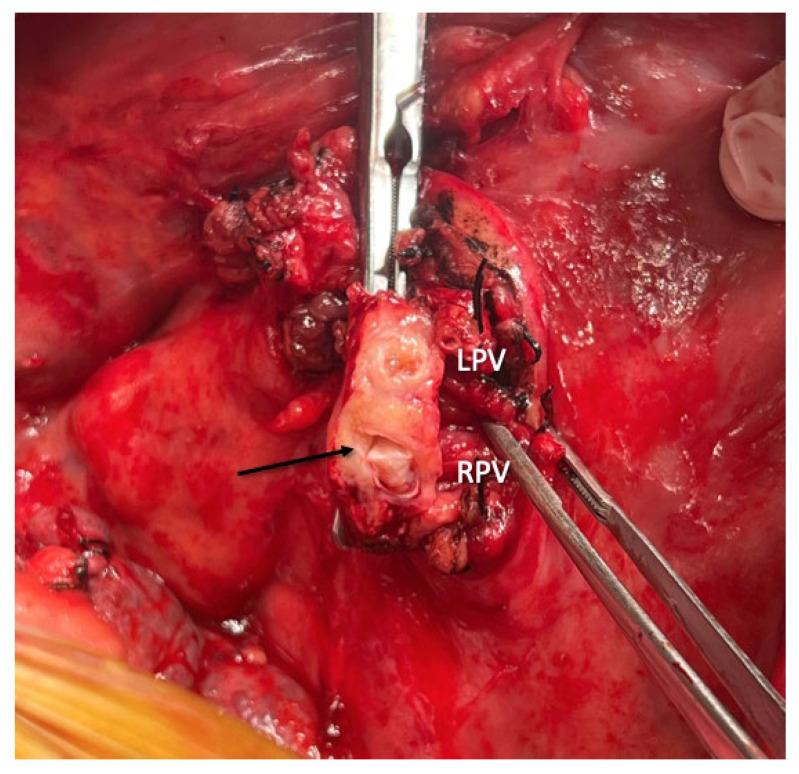
Portal vein clamped and transected at bifurcation level with identification of left (LPV) and right portal vein (RPV) with thrombus inside and identification of the plane between the endothelium and thrombus (black arrow).

This is the standard technique for PVT removal during transplantation, usually applied in grade 1 and 2 thromboses. After clamping and transecting the portal vein near its bifurcation, the vein wall is carefully retracted to visualize the plane between the endothelium and the thrombus. Once the thrombus is grasped, dissection and release are performed inferiorly until portal flow is reestablished (Figure 4) [22].

In cases with significant shunts or collaterals, ligation of these collaterals may be necessary to improve portal flow. When large splenorenal shunts are identified, the ligation of shunts should be prepared before implanting the liver graft. To achieve safer and quicker access to the shunt vessels, the ligation of left renal vein is considered as an alternative to ligating splenorenal shunt. These portal flow modulations should be prepared before implantation of the liver graft [22].

### 5.2. Jump Graft from SMV

In cases of total thrombosis where eversion thrombectomy is not feasible or when, despite thrombectomy, adequate portal flow cannot be restored in the absence of shunts or collaterals, but the superior mesenteric vein (SMV) remains preserved, a viable option is creating a jump graft from the SMV. By dissecting the SMV at the mesenteric root sufficiently to allow safe clamping, an interposition graft using a cadaveric venous graft (such as the iliac vein or internal jugular vein) or even synthetic prostheses can be performed, followed by a side-to-end anastomosis [22].

### 5.3. Other Strategies

Other alternatives are described and generally applicable in cases of extensive thrombosis when thrombectomy or graft interposition is not feasible. These approaches are usually associated with higher complication rates and worse survival compared to conventional techniques [26].

**Figure 5:**
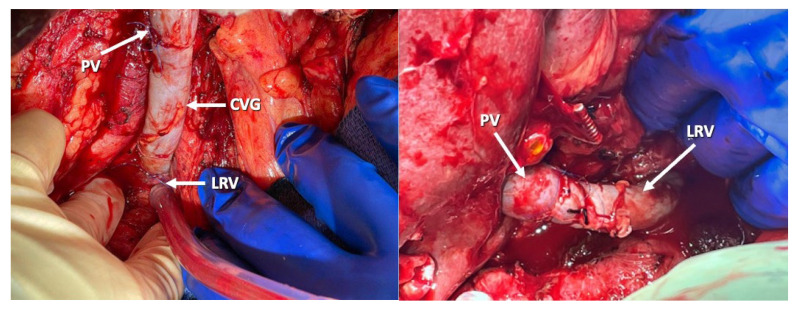
Renoportal Anastomosis. Legends: PV—Recipient’s Portal Vein; CVG—Cadaveric Venous Graft; LRV—Left Renal Vein.

Among them, renoportal anastomosis (RPA) between the recipient’s left renal vein and the graft’s portal vein is feasible alternative in the presence of a splenorenal shunt, as it allows for the maintenance of a more physiologic splanchnic circulation. However, this is a highly complex approach that may impact the recipient’s renal function. A meta-analysis including a total of 66 patients who underwent RPA during liver transplantation showed a 18.1% incidence of transient post-transplant renal dysfunction and a 3% incidence of chronic renal dysfunction, with overall patient and graft survival around 80% [22,27] (Figure 5).

### 5.4. Multivisceral Transplantation

Multivisceral transplant (MVT) is a backup option for liver transplant candidates with extensive portal vein thrombosis (Yerdel grade 4) and should only be managed at liver transplant centers experienced in handling such complex and high-risk patient profiles. A coherent contingency surgical plan must always be in place for these patients at the time of listing for liver transplantation. If robust portal venous flow is not established intraoperatively during LT, the surgical team must be prepared to employ alternative surgical techniques [28].

**Figure 6:**
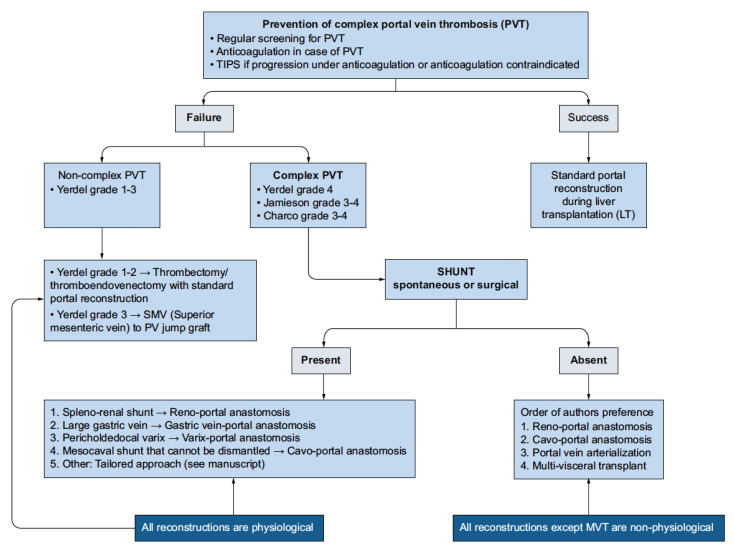
Bhangui algorithm for the treatment of non-malignant portal vein thrombosis during liver transplantation. Adapted from Bhangui P, Lim C, Levesque E et al., 2019 [29].

Bhangui proposed a highly detailed and sophisticated algorithm that attempts to address both issues, but this system has yet to achieve widespread use (Figure 6. Bhangui PVT Algorithm). According to Bhangui’s classification, Yerdel grade 4 PVT with the presence of spontaneous and/or surgical shunts should allow the surgeon to perform some form of physiological reconstruction. If no shunts are present, then the only form of physiological reconstruction for Yerdel grade 4 PVT is MVT [29].

Other non-physiological reconstructions, such as cavopoportal hemitransposition, or portal vein arterialization, are possible in this patient group, but will not fully decompress the splanchnic system [28].

A multivisceral transplant (MVT) involves a deceased donor, with a complete MVT graft including the stomach, pancreatic–duodenal complex, small intestine, and sometimes the right colon, as a cluster. The procedure is technically similar to a standard liver transplant. The decision to proceed with MVT is generally made preoperatively, based on detailed imaging and evaluation, particularly when there is extensive portal vein thrombosis that precludes restoration of adequate portal flow by conventional means [28].

If portal vein flow can be established through thrombectomy or a venous bypass graft, isolated liver transplantation may still be feasible, allowing transplantation of the other abdominal organs only if needed. However, if preoperative evaluation demonstrates that adequate portal vein inflow cannot be achieved, the recipient is scheduled for multivisceral transplantation, which includes resection of the liver, pancreas, duodenum, small intestine, right colon, and possibly the stomach, followed by transplantation of the multivisceral cluster graft [28].

Arterial inflow generally comes from the aorta or native celiac trunk, with venous flow through the portal vein and liver, exiting via hepatic veins. Both bicaval and piggyback approaches for the vena cava show similar results. Enteric anastomoses involve the stomach or proximal jejunum, ending with a terminal stoma or distal colon anastomosis [28].

MVT is a physiological treatment for Yerdel grade 4 portal vein thrombosis, replacing the entire portomesenteric system with functionally normal transplanted organs. It should be planned as a backup option for these cases. Furthermore, preoperative visceral artery embolization should be considered as a valuable adjunct in complex multivisceral transplant (MVT) candidates with cirrhosis, extensive portomesenteric vein thrombosis (PVT), and a hostile abdomen. By selectively occluding arterial inflow to the native viscera, the potential exists to mitigate life-threatening hemorrhage during the exenteration phase. Distal embolization, particularly utilizing Gelfoam, appears to confer a superior safety profile compared to proximal techniques, minimizing the risks of distal migration and subsequent systemic sequelae [28,30].

## 6. Post-Transplant Management

### 6.1. Anticoagulation in Post-Transplant Patients

Although thromboprophylaxis is well-established as a measure to reduce the risk of deep vein thrombosis (DVT) after major abdominal and pelvic surgeries, it is still unclear if the same strategy will reduce the incidence of DVT or portal vein thrombosis (PVT) in patients post-liver transplantation. Furthermore, this strategy may increase the risk of post-transplant morbidity and mortality due to bleeding complications from anticoagulation. Consequently, antithrombotic prophylaxis is not standard practice after liver transplantation, and currently, no established protocols guide the use of chemical thromboprophylaxis therapy in the post-transplant period [31].

Heparin is used to reduce the incidence of hepatic artery thrombosis after liver transplantation, primarily by increasing antithrombin III activity and affecting platelets and thrombin. In cases of cirrhosis or liver failure, the synthesis of coagulation factors, including antithrombin, is compromised, affecting the accuracy of anti-Xa tests and activated partial thromboplastin time for monitoring unfractionated heparin. These tests have limitations, underestimating heparin levels or overestimating its anticoagulant effect. Additionally, heparin-induced thrombocytopenia is a risk, although rare in liver recipients, except in Budd-Chiari syndrome patients. Monitoring platelet count and possible thromboses is vital when therapy is prolonged or there is a history of heparin use. Despite these limitations, unfractionated heparin is advantageous for liver transplant recipients with renal failure due to its favorable pharmacokinetics, rapid action, low bioavailability, and reversibility in case of bleeding complications. Due to the limitations of conventional tests in estimating plasma levels, the thrombin generation test emerges as an alternative that can elucidate the true anticoagulant effect of the drug [32].

A randomized controlled clinical trial by Xie and colleagues evaluated the efficacy and safety of thromboprophylaxis with enoxaparin versus normal saline in patients undergoing deceased donor liver transplantation. A total of 462 patients were recruited at two centers in China. The results indicated that prophylactic enoxaparin did not significantly reduce the incidence of PVT and DVT, even in high-risk patients, but increased the risk of major bleeding [31].

Direct oral anticoagulants (DOACs) are considered a promising alternative to heparin and low molecular weight heparin for preventing thrombotic complications after transplants, being safe in patients with cirrhosis due to their oral administration mode, fixed dose, and no need for laboratory monitoring. They act independently of antithrombin, which may be compromised in cirrhotic and is essential for the efficacy of low molecular weight heparin. However, they lack studies in post-liver transplant patients. With limited clinical experience, their anticoagulant effect is not easily reversible, and their elimination depends on liver and kidney function, with the risk of excessive accumulation and bleeding in the postoperative period. In transplant patients, who often have compromised kidney and liver function, these factors limit the interest of direct oral anticoagulants for thromboprophylaxis shortly after surgery, in the immediate postoperative period [32].

A retrospective study conducted by Bos et al. (2021) [33] at two medical centers evaluated whether therapeutic dose anticoagulant administration after liver transplantation is beneficial in preventing recurrence of PVT in patients who had Yerdel grade I or II portal vein thrombosis before transplantation. The results indicated that using therapeutic dose anticoagulants did not reduce the recurrence of PVT and was associated with a significantly higher risk of hemorrhagic events and a longer hospital stay, without improving graft or patient survival. Therefore, the study concluded that therapeutic dose anticoagulants are unnecessary to prevent the recurrence of grade I or II PVT after liver transplantation and may cause more harm than benefit [33].

The study conducted by Yeo et al. (2022) [34] assessed the impact of pre-transplant PVT on postoperative outcomes, specifically addressing the role of anticoagulant management after liver transplantation. The study highlights that pre-operative PVT, especially in higher grades (grades 3 and 4), significantly increases risks of overall mortality and graft failure. Moreover, this condition is strongly associated with a higher likelihood of developing postoperative PVT, being more than five times greater. Although waitlist mortality was not significantly impacted by pre-transplant PVT, the study suggests that post-transplant prophylactic anticoagulation could be beneficial. This approach could potentially reduce the risk of new thrombosis and improve survival rates, especially among patients with high-grade pre-transplant PVT (grades 3 and 4) [34].

Therefore, due to the lack of a coagulation test that reliably predicts the risk of bleeding or thrombosis in the perioperative period of liver transplantation, the use of viscoelastic tests such as thromboelastography (TEG) or rotational thromboelastometry (ROTEM) has been explored to provide a more comprehensive assessment of coagulation status. While TEG can offer real-time information and help guide transfusion and anticoagulation strategies intraoperatively, its role in predicting perioperative bleeding or thrombosis risk remains limited and is not yet standardized in clinical practice. For this reason, it is not possible to make solid recommendations about thromboprophylaxis in this context, especially in patients with PVT before liver transplantation. More well-designed clinical studies are needed to evaluate the efficacy, safety, and tolerability of anticoagulant drugs for preventing thrombotic events in transplanted patients [32].

### 6.2. Endovascular Management of Portal Vein Thrombosis

There are various approaches to catheterization of the portal vein. The percutaneous transhepatic approach is the conventional method for accessing the portal venous system and has been widely used for endovascular interventions [35].

However, in the post-transplant setting, this technique may be limited by factors such as altered anatomy, postoperative fibrosis, hematoma, and ascites. These conditions can serve as relative contraindications or may be exacerbated by percutaneous puncture of the portal vein, increasing the risk of complications, as highlighted in recent case series [35].

The transjugular approach, commonly used for TIPS, is a significant alternative, especially for patients with portal hypertension and variceal bleeding. However, this technique may not be feasible if central intrahepatic portal vein branches are occluded or if there is a large or infiltrative malignancy along the puncture path, due to increased risk of tumor seeding [35].

**Figure 7:**
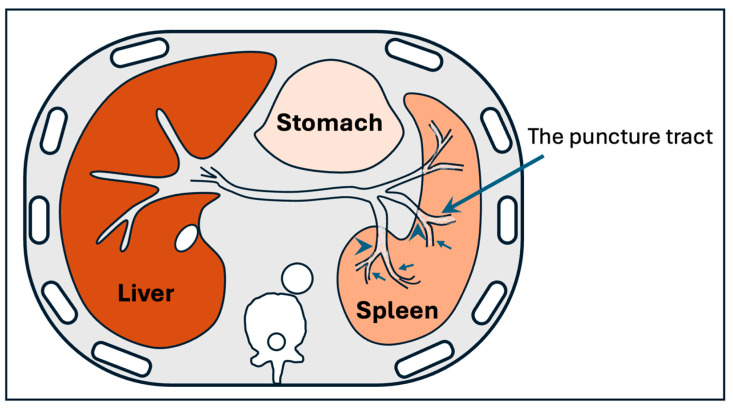
Anatomical illustration of the splenic vein and its branches. The first branch of the splenic vein (arrowheads) is surrounded by the splenic parenchyma and segmental splenic vein (arrows). Adapted from Zhu K, Meng X, Zhou B et al., 2012 [36].

Alternative routes, such as the transmesenteric approach, require a minilaparotomy and are more invasive. The percutaneous transsplenic (Figure 7) approach is another option, particularly in complex or post-transplant cases. While effective, transsplenic access is associated with a higher risk of bleeding, including the development of perisplenic hematoma, pseudoaneurysm, and intrasplenic arteriovenous fistula, and should be reserved for selected patients where other routes are not possible [35,36].

**Figure 8:**
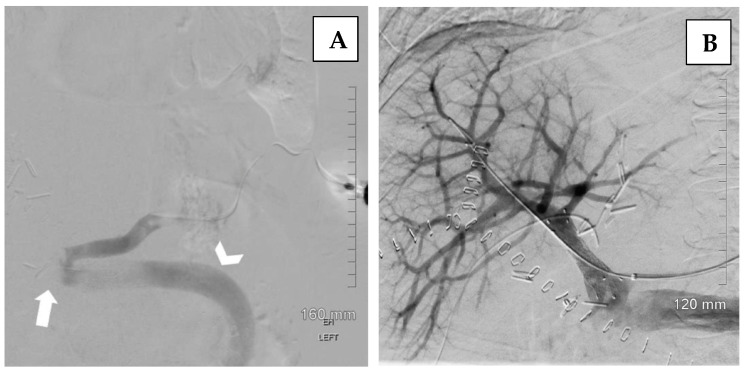
Illustration of an exemplary case of a transsplenic approach in managing portal vein thrombosis following liver transplantation. (**A**)—Splenic venography demonstrates complete occlusion of the main portal vein of the transplant (arrow) with a large diameter portosystemic shunt associated (arrowhead). (**B**)—Post-stent placement venography shows improved blood flow through the main portal, left and right portal, and intrahepatic veins, with no evidence of portal vein thrombus or residual stenosis. Persistent opacification of the large portosystemic shunt remains. Adapted from Brown MA, Donahue L, Gueyikian S, Hu J, Huffman S, 2020 [35].

**Figure 9:**
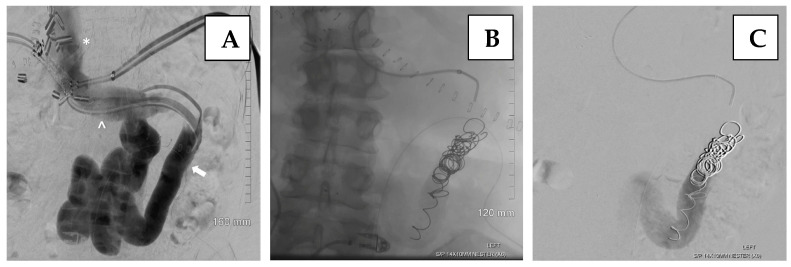
Illustration of an exemplary case of a transsplenic approach in managing portal vein thrombosis following liver transplantation. (**A**)—Venography demonstrates a splenorenal shunt (arrow), along with a mildly dilated inferior vena cava (*) and left renal vein (^). (**B**)—Venography depicts multiple coils used to embolize the splenorenal shunt following the recanalization of the main portal vein. (**C**)—Venography shows decreased flow through the splenorenal shunt after coil embolization. Adapted from Brown MA, Donahue L, Gueyikian S, Hu J, Huffman S, 2020 [35].

The transsplenic approach offers a direct pathway to access the portal venous system (Figure 8), as well as associated varices or shunts, while avoiding potential injury to the transplanted liver that may occur with transhepatic or transjugular approaches. Transsplenic recanalization of an occluded portal vein through angioplasty and stenting with shunt embolization can be a safe and effective treatment alternative in adult liver transplant recipients (Figure 9) [36].

**Figure 10:**
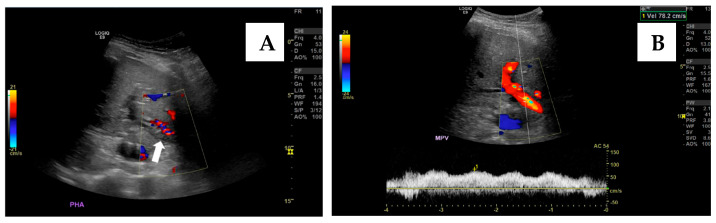
Illustration of an exemplary case of a transsplenic approach in managing portal vein thrombosis following liver transplantation. (**A**)—Hepatic ultrasound in a late postoperative period shows complete occlusion of the main portal vein (arrow). (**B**)—Hepatic ultrasound on the first day after the radiological intervention demonstrates a patent portal venous system following portal stent placement. Doppler analysis and waveform show improved hepatopetal flow velocities. Adapted from Brown MA, Donahue L, Gueyikian S, Hu J, Huffman S, 2020 [35].

The effectiveness of the transsplenic approach can be clearly confirmed by Doppler ultrasound findings. In this case, complete absence of portal venous flow was observed before the intervention (Figure 10A), while restoration of hepatopetal flow was demonstrated after angioplasty and stenting, confirming technical and clinical success (Figure 10B). These ultrasound images highlight the pivotal role of US not only in diagnosis, but also in documenting treatment efficacy in real time [35].

Endovascular transsplenic recanalization with stenting and shunt embolization is a viable method for treating main portal vein thrombosis in an adult liver transplant recipient. A transsplenic approach can be an effective alternative to a transhepatic approach in the context of portal vein complications post-liver transplantation [36].

### 6.3. Late Portal Vein Thrombosis After Liver Transplantation

Late portal vein thrombosis (PVT) is defined as thrombosis occurring more than three months after liver transplantation. Although it is less frequent than early or perioperative PVT, late post-transplant PVT can have significant clinical implications. It may remain asymptomatic for a long period or present with signs of portal hypertension, such as gastrointestinal bleeding or ascites. Risk factors include persistent low portal flow, anastomotic stenosis, recurrence of the primary disease, or technical complications from the transplant procedure. Diagnosis is typically based on Doppler ultrasound or cross-sectional imaging. Management depends on the degree of thrombosis and clinical presentation and may involve anticoagulation, endovascular interventions (such as angioplasty or stenting), and, in selected cases, surgical revision. Early identification and individualized treatment are important to improve graft and patient outcomes [26,32].

## 7. Conclusions

Portal vein thrombosis (PVT) poses significant challenges in liver transplantation (LT), affecting both clinical and surgical approaches due to its high prevalence in patients with chronic liver disease. Effective identification and management of PVT during pre-transplant evaluation are crucial, with awareness of the coagulation anomalies that drive its development. The Yerdel classification aids in surgical planning by informing strategies such as anticoagulant use and interventions like TIPS to prevent thrombus progression. Procedures such as thrombectomy and the use of grafts are tailored to the severity of the thrombosis. Specific surgical techniques, including eversion thrombectomy or jump grafts from the superior mesenteric vein, are applied according to the thrombus extent.

In the post-transplant setting, the use of anticoagulants remains a delicate issue due to bleeding risks, although some studies indicate DOACs as promising alternatives. While aspirin shows potential for preventing hepatic artery thrombosis, the efficacy of therapeutic doses of post-transplant anticoagulants remains debated, suggesting an increased risk of bleeding. Considering the lack of reliable tests to predict thrombotic and hemorrhagic risks, thromboprophylaxis recommendations remain complex, especially in pre-transplant contexts. Therefore, future research should focus on robust clinical trials to evaluate anticoagulant treatments, aiming to improve transplant survival and outcomes.

## Figures and Tables

**Table 1:** PVT classification proposed by Yerdel.

Grade	Description
Grade I	<50% of vessel lumen thrombosis of PV, with or without minimal extension to the SMV
Grade II	>50% occlusion of the PV, including total occlusions, with or without minimal extension to the SMV
Grade III	Complete thrombosis of both PV and proximal SMV (distal SMV is open)
Grade IV	Complete thrombosis of the PV as well as proximal and distal SMV

PVT: portal vein thrombosis; PV: portal vein; SMV: superior mesenteric vein.

## Abbreviations

PVT	Portal Vein Thrombosis
LT	Liver Transplantation
TIPS	Transjugular Intrahepatic Portosystemic Shunts
MVT	Multivisceral Transplantation
